# A census of actin-associated proteins in humans

**DOI:** 10.3389/fcell.2023.1168050

**Published:** 2023-04-28

**Authors:** Iyer Meenakshi S, Madan Rao, Satyajit Mayor, Ramanathan Sowdhamini

**Affiliations:** ^1^ National Centre for Biological Sciences, TIFR, Bangalore, India; ^2^ Molecular Biophysics Unit, Indian Institute of Science, Bangalore, India; ^3^ Institute of Bioinformatics and Applied Biotechnology, Bangalore, India

**Keywords:** actin-binding proteins, actin-associated proteins, cytoskeleton, protein–protein interactions, calponin homology domain

## Abstract

Actin filaments help in maintaining the cell structure and coordinating cellular movements and cargo transport within the cell. Actin participates in the interaction with several proteins and also with itself to form the helical filamentous actin (F-actin). Actin-binding proteins (ABPs) and actin-associated proteins (AAPs) coordinate the actin filament assembly and processing, regulate the flux between globular G-actin and F-actin in the cell, and help maintain the cellular structure and integrity. We have used protein–protein interaction data available through multiple sources (STRING, BioGRID, mentha, and a few others), functional annotation, and classical actin-binding domains to identify actin-binding and actin-associated proteins in the human proteome. Here, we report 2482 AAPs and present an analysis of their structural and sequential domains, functions, evolutionary conservation, cellular localization, abundance, and tissue-specific expression patterns. This analysis provides a base for the characterization of proteins involved in actin dynamics and turnover in the cell.

## 1 Introduction

All eukaryotes depend on the actin cytoskeleton to provide a framework to support the internal movements of proteins, organelles, and vesicles across the cell. Actin participates in many cellular functions, including cell division, maintaining cellular structure and motility, intracellular transport, transport of cargo across the cell, and muscle contraction (Dominguez and Holmes, 2011). More recently, actin at the membrane cortex in conjunction with myosin has been shown to drive nano and mesoscale organization of cell membrane proteins (Fritzsche et al., 2017). Actins are one of the most abundant proteins in the cell, with a slow turnover rate of weeks in muscle cells, and are generally ubiquitously expressed, which mirrors their central and conserved role in gene regulation (Pollard, 2016).

Actin can dynamically interchange between two states: globular monomeric (G) actin and filamentous polymeric (F) actin. This process occurs by actin polymerization at the barbed end of the actin filament and by depolymerization of actin from the pointed end of the filament. The balance between the rates of polymerization and depolymerization decides the state of the actin filament (Fujiwara et al., 2018). Many actin-binding proteins regulate these steps and are, in turn, affected by cellular signals such as pH, phosphorylation status, calcium, and phosphoinositides (Senju et al., 2017; Varland et al., 2019). Actin is also one of the most conserved proteins across eukaryotes (Van Troys et al., 1999). This conservation is attributed to the need for actin monomer (G-actin) to bind to itself to self-assemble into polymers and also to interact with a plethora of actin-binding regulatory proteins or proteins using this assembly as a scaffold (Pollard, 2016).

Many actin-binding proteins have been identified and studied across eukaryotes (Ayscough, 1998; Van Troys et al., 1999; Dos Remedios et al., 2003; Lappalainen, 2016; Pollard, 2016). These proteins together maintain a pool of actin monomers available for polymerization by regulating all aspects of actin assembly. These proteins have been classified based on the functions such as nucleation (e.g., ARP2/3, Spire, and formins), cross-linking or bundling actin filaments (e.g., fimbrin, filamin, villin, and α-actinin), regulation of filament assembly and disassembly (e.g., profilin, thymosin β_4_, ADF/cofilin, gelsolin, and capping protein) involved in the formation of supramolecular structures, for example, focal adhesions (e.g., vinculin, talin, paxillin, and integrin), membrane interactions (e.g., ezrin), and proteins that help transport cargo over actin filaments (e.g., myosin) (Dos Remedios et al., 2003). However, certain proteins perform multiple functions, and it is difficult to classify them into such functional categories (Van Troys et al., 1999). Formins nucleate actin filaments and promote barbed end polymerization, and some formins can also bundle actin filaments (Valencia and Quinlan, 2021). The gelsolin family of calcium-regulated ABPs is involved in severing actin filaments and also acts as capping proteins that bind to barbed ends of actin filaments (Burtnick et al., 2004). Actin-associated proteins (AAPs) interact with actin-binding proteins to mediate the various cellular functions of actin. For example, nucleation-promoting factors such as Wiskott–Aldrich syndrome protein (WASp) and cortactin associate with the actin-binding protein APR2/3 and activate the complex to promote nucleation of actin filaments (Ji et al., 2020; Romero et al., 2020).

There are 32 actin family members in humans, and these are categorized into five classes, conventional actins, actin-related proteins (Arp) (conventional Arps, actin-like proteins, and alpha- and beta-centractins), and POTE (prostate ovary testis expressed protein) ankyrins E, F, I, J, and K. Conventional actins are major constituents of the cell cortex and the cytoskeleton, and the centractins are associated with the centrosome during cell division and the Golgi apparatus (Fouquet et al., 2000). The actin-related proteins are conserved across eukaryotes and are involved in actin filament branching and chromatin remodeling, and are components of the dynactin complex (Goodson and Hawse, 2002). The actin-like proteins are present in mammals and are mostly tissue-specific (Chen et al., 2023; Ferrer et al., 2023). The POTE ankyrins have a tissue-specific expression, and some of these proteins are highly expressed in cancer cells (Bera et al., 2004; Liu et al., 2009).

In this work, we focus on conventional actins and their associated proteins. Vertebrates have three main groups of functional highly conserved conventional actin isoforms: viz. alpha, beta, and gamma actins. These are encoded by six genes and are classified into four muscle actins and two non-muscle actins. The muscle actins (alpha actins) are grouped into skeletal muscle α-actin, vascular smooth muscle α-actin, enteric smooth muscle actin, and cardiac muscle α-actin. Non-muscle cells have two isoforms of cytoplasmic actin (ß-actin and γ-actin) (Arora et al., 2023). The actin isoforms have subtle differences in the ATP-binding site, actin-dimerization site, and the interaction interface which is used in the interaction with other proteins. As a result, they display differences in biochemistry, filament stability, and interactions with actin-binding proteins. These differences may result in the formation of actin cytoskeletal networks with distinct dynamics and compositions (Arora et al., 2023).

Recent studies have explored the protein–protein interaction landscape of the human proteome. Several databases have integrated PPI data from different sources, such as high-throughput co-immunoprecipitation studies, pull-down assays, yeast two-hybrid assays, computational prediction methods, and literature (Orchard et al., 2014; Chatr-Aryamontri et al., 2017; Szklarczyk et al., 2019). This has enabled us to identify interacting partners of the proteins and the complexes they are a part of and construct organism-wide protein function association networks (Cusick et al., 2005; Stelzl et al., 2005; Li et al., 2010). Initiatives such as the Gene Ontology (GO) resource assign functions to genes with several levels of evidence (Gene Ontology Consortium, 2015). Several literature reports are also available for actin-binding proteins discovered in several organisms using biochemical experiments. The three-dimensional structures of a few such interacting protein domains complexed with actin are also available. In addition, there are some protein domain families known in nature which have been implicated in actin-binding, such as vinculin, villin headpiece domain, and gelsolin domain (Pollard, 2016). However, a global census that systematically lists the number of proteins that bind or associate with actin is currently not available. We integrate the metadata from resources describing protein–protein interactions, protein domain family databases, the GO resource, and literature mining to identify and create a resource of putative AAPs. From here on, we refer to both the actin-binding and actin-associated proteins as AAPs.

Here, we present a review of 2,482 likely human AAPs along with the actin-binding binding domains and motifs for some of them. We also present a functional domain characterization of the AAPs and their evolutionary conservation, cellular localization, expression patterns, and predicted functions. These predicted AAPs underlie many different aspects of human physiology, disease, and variation, highlighting the importance of these proteins. We hope that this will guide future system-wide studies of AAPs.

## 2 Methods

### 2.1 Annotation of the human proteome

The list of the reviewed (Swiss-Prot) human proteins with details of Gene Ontology terms and subcellular localization post-translational modifications was downloaded from UniProt (https://www.uniprot.org/) (release 2022_2) (The UniProt Consortium, 2021).

The corresponding reviewed human protein FASTA sequences along with the isoforms were also retrieved from UniProt. Hmmscan (HMMER suite v3.1b2) was used to assign Pfam family domains (Pfam A HMM v36) with an E-value of 0.01 and gather score cutoff to identify domains in the human proteome (Eddy, 2011; Mistry et al., 2021). Domain architectures were assigned to protein sequences using an in-house Python script (Iyer et al., 2018). An independent E-value threshold of 0.001 and a model coverage filter of 0.7 were used, and domain overlaps of up to 25 residues were allowed. In the case of multiple Pfam family HMM aligning to the same region in a sequence, the HMM with the lowest independent E-value was given preference.

### 2.2 Identification and annotation of putative AAP

The dataset of proteins has three lines of evidence of actin-binding, namely, 1) actin-binding detected by PPI databases, 2) the presence of at least one canonical actin-binding domain, 3) literature, and 4) Gene Ontology (GO) annotation-based identification.

#### 2.2.1 Databases with protein–protein interaction data

A list of human actins was created from the UniProt human proteome consisting of six reviewed proteins (Table 1). The protein interacting partners of these proteins were searched for in protein–protein interaction databases using the UniProt/Ensembl identifiers (The UniProt Consortium, 2021).

**TABLE 1:** List of actins used in the study.

Entry	Protein names	Gene names	Cellular localization	Tissue enriched	Length
P62736	Actin, aortic smooth muscle (alpha-actin-2)	ACTA2, ACTSA, ACTVS, and GIG46	Cytoskeleton	Smooth muscle cells and myoepithelial cells	377
P68032	Actin, alpha cardiac muscle 1 (alpha-cardiac actin)	ACTC1 and ACTC	Cytoskeleton	Heart, and skeletal and smooth muscle	377
P68133	Actin, alpha skeletal muscle (alpha-actin-1)	ACTA1 and ACTA	Cytoskeleton	Myoepithelial cells, heart, and skeletal and smooth muscles	377
P63261	Actin, cytoplasmic 2 (gamma-actin)	ACTG1 and ACTG	Cytoskeleton	Ubiquitously expressed	375
P63267	Actin, gamma-enteric smooth muscle (alpha-actin-3) (gamma-2-actin)	ACTG2 and ACTA3 ACTL3 and ACTSG	Cytoskeleton	Myoepithelial cells	376
P60709	Actin, cytoplasmic 1 (beta-actin)	ACTB	Cytoskeleton	Ubiquitously expressed	375

The UniProt identifiers, gene names, protein length, and protein localization details of the six different actins used in the study have been provided. The cellular localization and tissue enrichment were derived from the UniProt and HPA database.

We used the STRING, BioGRID, DIP, MINT, HitPredict, HINT, mentha, HuRI, and HIPPIE databases for extracting the PPI interacting partners of the six reviewed actins. The links of these resources with the interaction score thresholds are provided in Table 2. We chose only the physical interactions with high interaction scores from the datasets. This was done to ensure that only high-confidence interactions were included and to remove the background interactions.

**TABLE 2:** List of protein–protein interaction works used in the study.

Resource	Version/access date	File	Link	Thresholds
STRING	v11.5	9606.proteins.links	https://string-db.org/	Interaction score >700
BioGRID	24 June 2022	Keyword search with UniProt ids	https://thebiogrid.org/	Score >0.6
DIP	v2017	Hsapi20170205.txt	https://dip.doe-mbi.ucla.edu/dip/	Has only high-confidence interactions
HINT	29 June 2022	Keyword search with UniProt ids	http://hint.yulab.org/	
HuRI	28 June 2022	HI-union.tsv	http://www.interactome-atlas.org/	
IntAct	28 June 2022	Keyword search with UniProt ids	https://www.ebi.ac.uk/intact/	Score >0.6
MINT	24 June 2022	Human data	https://mint.bio.uniroma2.it/	Score >0.6
mentha	28 June 2022	*Homo sapiens* data	http://mentha.uniroma2.it/	Score >0.6
HitPredict	28 June 2022	Keyword search with UniProt ids	http://www.hitpredict.org/	Annotation score >0.5; method-based score >0.485
HIPPIE	30 June 2022	Keyword search with UniProt ids	http://cbdm-01.zdv.uni-mainz.de/∼mschaefer/hippie/	Score >0.73

The list of the protein–protein interaction web servers used in the study and the links have been listed. The thresholds used for filtering high-confidence interactions and the file names for the species data (human) have also been listed. For some of the webservers, we used a keyword search of the protein UniProt ids (Table 1) to query for the interacting proteins.

In addition, the PDB database (https://www.rcsb.org/) was searched using the keyword actin-binding, and the actin-interacting protein names were extracted from the results (Burley et al., 2021). This list was manually curated for actin-binding proteins, and such proteins were added to the list.

#### 2.2.2 Proteome-wide scan for Pfam actin-binding families

A list of actin-binding domains was created using the information available from the literature and the Pfam database (https://pfam.xfam.org/) to associate the actin-binding function (Supplementary Table S1A) (Mistry et al., 2021). We extracted the proteins having Pfam domains associated with the GO terms related to actin-binding, such as “actin monomer binding” and “actin cytoskeleton” from the Pfam2GO mapping file (pfam2go.txt) (Supplementary Table S1B) (Forslund and Sonnhammer, 2008). The Pfam2GO mapping file provides GO annotations, where available, for the Pfam domain families. Proteins with at least one known actin-binding domain were identified from their domain architecture and annotated as AAPs. The procedure used for Pfam domain annotation of the human proteins is described in Section 2.1.

#### 2.2.3 GO-term-based information mining from databases

We created a list of GO terms associated with actin-binding from the annotations provided in the Gene Ontology database (http://geneontology.org/) (Supplementary Table S1C). The annotation file was downloaded from the link: http://geneontology.org/docs/download-go-annotations/, and the GO terms and identifiers related to actin-binding were extracted (Gene Ontology Consortium, 2015). The GO terms are categorized into biological process, cellular component, and molecular function (Ashburner, 2000). We derived the functional annotation metadata for all human proteins from UniProt and extracted the proteins annotated with the GO terms in the above list. This constituted the list of putative actin-binding and actin-associated human proteins derived from GO functional annotations.

In addition, literature mining was carried out to identify sequences that have been shown to bind actin. We manually added these proteins to the AAP list.

### 2.3 Identification of protein domains in the human AAPs

The AAPs were classified into Pfam domain families and SCOP structural families based on their domain composition (Mistry et al., 2021; Chandonia et al., 2022). The Pfam domain families were assigned as described in Section 2.1. The Pfam clans were assigned to the sequences using the Pfam clan mapping file from the Pfam database (PfamC.txt).

The SCOP domains were assigned using HMMs derived using structural alignments of SCOP superfamily members in PASS2.4 (Gandhimathi et al., 2012). HMMscan tool from the HMMER suite (HMMER suite v3.1b2) was used to assign the HMMs to the sequences (Potter et al., 2018). BlastP (NCBI-BLAST + v2.2.13) was used to carry out a sequence search against the PDB database (Camacho et al., 2009; Burley et al., 2021). UniProt and InterPro annotations were used to manually map the SCOP domain/classes to the remaining sequences (Blum et al., 2021; The UniProt Consortium, 2021). The information on coiled-coil regions, signal and transit peptides, and regions with disorders was also derived.

We also identified actin-binding residues, functional motifs, and domains where available from UniProt and the literature.

### 2.4 Cellular localization and tissue specificity analysis

The cellular localization of the AAPs was identified from the GO terms and subcellular location metadata from UniProt. The proteins with the annotations “intramembrane,” “topological domain,” or “transmembrane” metadata were annotated as integral membrane proteins. The peripheral membrane proteins were identified using the “subcellular localization” metadata from UniProt. The proteins with lipid modifications were identified using “lipidation” metadata and added to the list of peripheral membrane proteins.

These data were supplemented using the subcellular location and tissue expression data from the Human Protein Atlas (HPA) database (https://www.proteinatlas.org/) (Uhlén M et al., 2015). We retained the terms with reliability scores “enhanced,” “supported,” and “approved” (filtered out the annotations with “uncertain” reliability) and the expression levels “high,” “medium,” or “low” (filtered out the annotations with “not detected” expression levels).

### 2.5 Evolutionary conservation of AAPs and Pfam domains in AAPs

The evolutionary conservation of AAPs was derived from the eggNOG database (http://eggnog-mapper.embl.de/) (Huerta-Cepas et al., 2019). An E-value threshold of 0.001 with a 50% minimum query and subject coverage was used. One-to-one and one-to-many orthologs were considered for the analysis. The database also provides the COG classification for the sequences (Galperin et al.,2021). The taxonomic distribution details of the Pfam domains identified in the AAPs were derived from the Pfam family details page (Mistry et al., 2021).

### 2.6 Enrichment analysis considering GO, disease, and pathway annotations

Enrichment analysis was carried out using UniProt protein ids using the ShinyGO webserver (v0.76.3) (Ge et al., 2020). An FDR cutoff of 0.001 was used. All the SwissProt protein ids from the human proteome were provided as the background protein list. The Kyoto Encyclopedia of Genes and Genomes (KEGG) pathway enrichment analysis was also carried out (Kanehisa et al., 2016). The disease-related information was gathered from the HPO and OMIM dataset provided in the webserver (Amberger et al., 2009; Köhler et al., 2021). Transcription factor-related information was derived from the RegNetwork module implemented in ShinyGO, and the list of enriched motifs in the promoter region of AAP genes and the TFs binding to these sites were recorded (Liu et al., 2015). The expression data of AAPs under pathology conditions were derived from HPA pathology data (Uhlén et al., 2015). The reliability scores and expression levels were filtered similarly as described in the section on cellular localization. We derived the cancer-related expression data for the AAPs from the HPA database using a prognostic-favorable association filter of >0.0001. We also used the disease annotation data from the DisGeNET webserver (v7.0) (https://www.disgenet.org/), with a semantic score filter of => 0.3 (Piñero et al., 2020).

**FIGURE 1:**
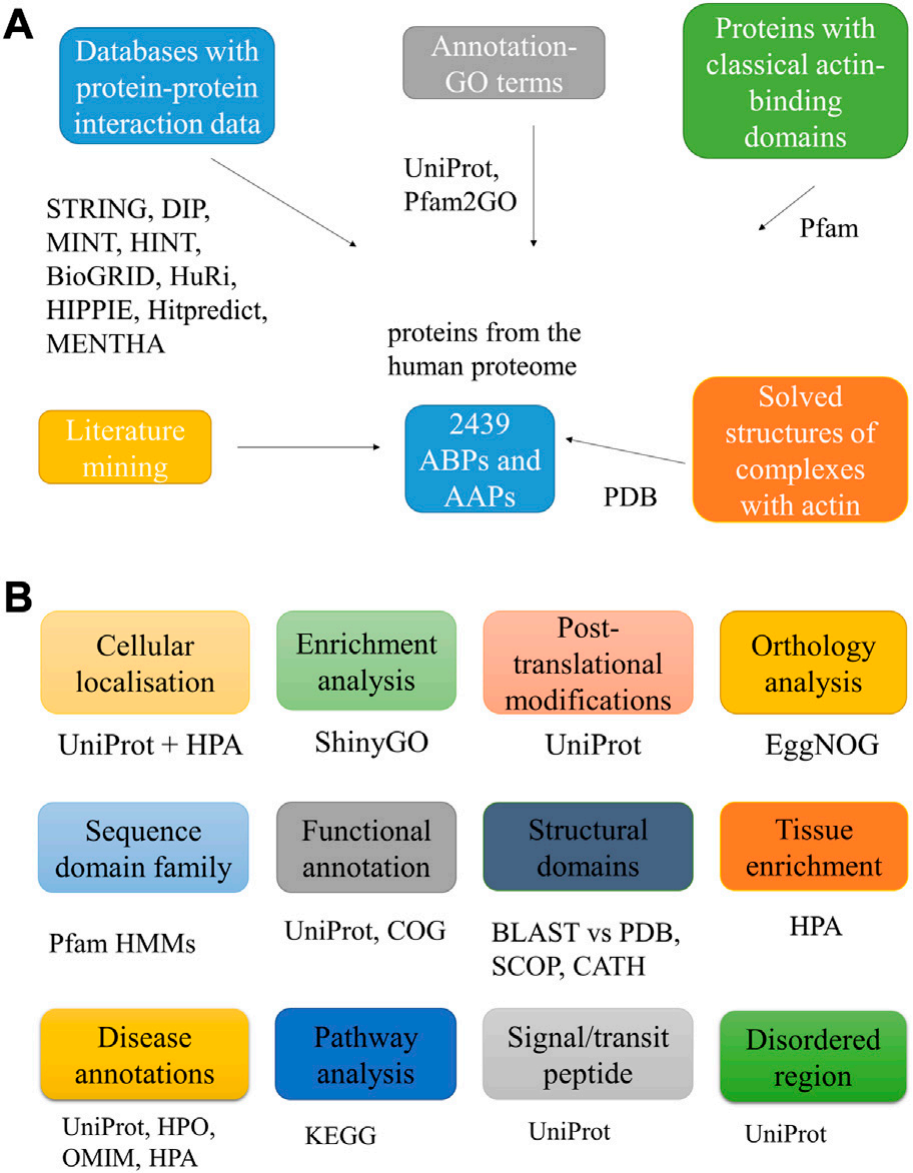
Workflow used to mine putative AAPs. The figure lists the different approaches used to identify the AAPs **(A)**. It also lists the various downstream analyses carried out for the proteins with the tools, databases, and web servers used for the same **(B)**.

Figure 1A schematically represents the methodology for mining the AAPs, and Figure 1B lists the downstream analysis and the tools used.

## 3 Results

Actin-binding and associated proteins have usually been classified according to their functional effects on actin organization, such as actin-nucleating proteins, capping proteins, and cross-linking proteins (Dos Remedios et al., 2003). A recent study reported 403 unique AAPs in vertebrates identified through literature mining (Gao and Nakamura, 2022). We have used approaches such as identifying interaction partners through databases, GO term annotations, and scanning the proteome for protein domains with actin function in addition to literature mining.

### 3.1 Establishing a census of AAPs

**FIGURE 2:**
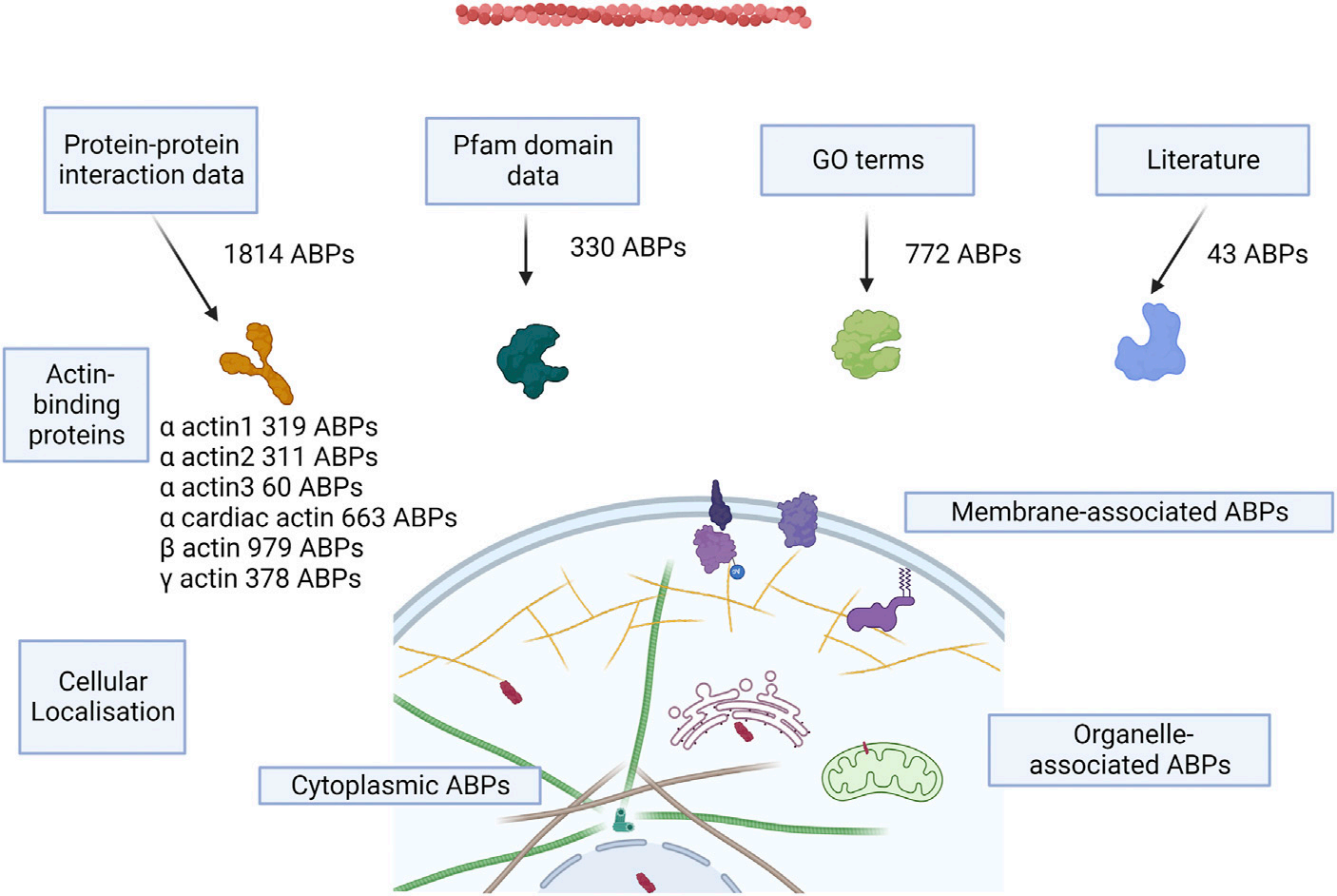
Concept figure. The figure shows a schematic of the approaches to annotate AAPs from the human proteome and the number of AAPs identified from each method. Most of the proteins are present in the cytoplasm, some of the proteins associate with the membrane, and few proteins are present in organelles like the nucleus, mitochondria, ER, and the Golgi bodies.

We identified actin-binding proteins from different methods—protein–protein interaction databases (Supplementary Data S1), UniProt GO annotations, Pfam GO annotations, Pfam domain survey, and literature survey (Supplementary Data S2). This resulted in a total count of putative 2,482 AAPs, which is roughly 10% of the human proteome (Figure 2), which formed the basis of subsequent analyses described here. This includes the proteins involved in maintaining the structural integrity of actin filaments, motor proteins, and the enzymes involved in the dynamics and turnover of actin in the cell. This list provides a starting point for future curation efforts. This catalog could change as experimental studies uncover new AAPs or identify that certain putative AAPs (with classical ABDs) have evolved to adopt new functionalities unrelated to actin binding. The protein–protein interaction network for the skeletal actin (alpha-actin 1) is provided in Supplementary Figure S1. The highest confidence interactions (score 0.9) and a maximum of 50 interactions were retained. The list of proteins is presented in Supplementary Data S3.

#### 3.1.1 AAPs derived from PPI databases

We used the protein–protein interactions listed for the six human actins across several databases and web resources (Table 2). We retained the high-confidence interactions using stringent interaction score thresholds for the protein pairs in the databases. This resulted in a cumulative set of 1814 proteins. Most of the interaction partners were for beta-actin (979), alpha-cardiac actin (663), and gamma-actin (378) (Figure 2). The isoform details of the AAPs are provided in the Supplementary Text (Section 1).

#### 3.1.2 AAPs derived from Pfam domain annotation

The Pfam database is a database of protein families that provides HMM models of protein domains for each family using a carefully curated seed alignment of the domain sequences. We used the Pfam protein-domain hidden Markov models (HMMs) to search the human proteome, comprising 20,386 protein-coding genes (21,989 additional isoforms), followed by stringent filtering criteria to assign domains to sequences. We defined proteins as AAPs if they contain Pfam domains known to directly bind actin, such as the CH domain and vilin headpiece domain (Supplementary Table S1A–B). We identified 243 AAPs (727 isoforms) from the literature-based Pfam domain assignment method.

#### 3.1.3 AAPs derived from GO metadata

The GO resource provides functional annotations which are manually curated from experimental evidence or computationally derived using approaches such as inferring from the function of homologs and orthologs. The GO annotation metadata have been integrated into several protein databases, such as UniProt, InterPro, Pfam, and PDB. We used the GO annotations of proteins in the UniProt database to identify 772 AAPs (2,178 isoforms). In addition, we used Pfam domain GO annotations (87 AAPs with 191 isoforms) and PDB GO annotations (27 AAPs with 61 isoforms) to identify protein domains with putative AAP functions (Supplementary Data S2).

We manually added AAPs that were missed by domain searches but are clearly defined in the literature (Supplementary Data S2). This includes proteins such as S100 calcium-binding protein A6 and cytoskeleton-associated protein 5, which have recently been shown to bind actin (Slater et al., 2019; Jurewicz et al., 2020).

### 3.2 Sequence domains in AAPs

**FIGURE 3:**
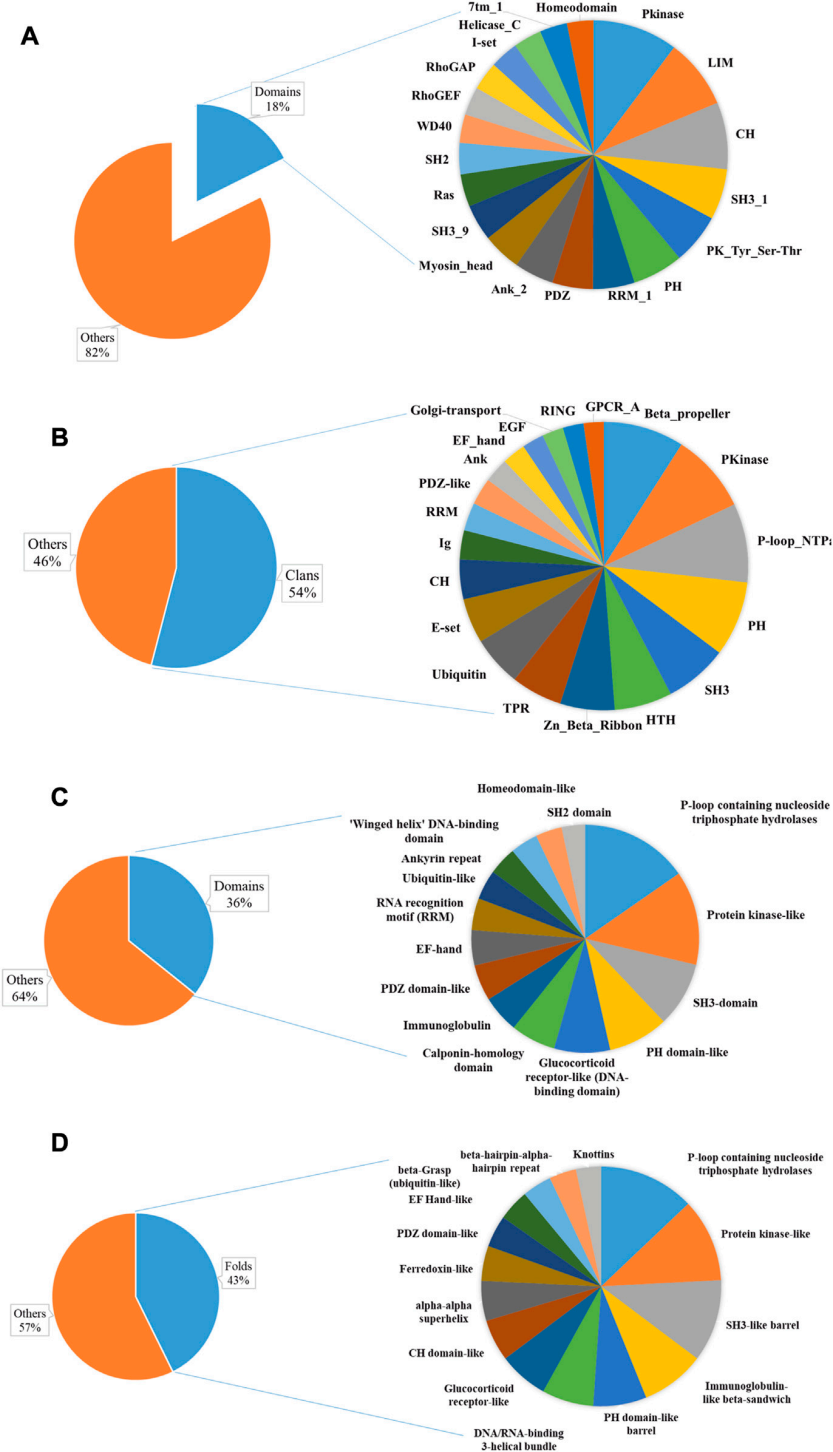
Sequence and structural domains in putative AAPs. The Pfam family **(A)** and clan **(B)** distribution have been plotted as a pie chart. The top 20 domain families represent only 18% of the total Pfam families, indicating diversity. The SCOP superfamily **(C)**, fold **(D)**, and class distribution have been plotted. This shows the diversity of actin-binding domains in nature.

The AAPs we identified contain a repertoire of 1820 distinct Pfam (functional) domains in 1,574 distinct domain architectures (1832 domains with 2,109 domain architectures including isoforms) (Supplementary Table S2). These Pfam families belong to 307 Pfam clans. Among the domain families, only seven are found in more than 40 proteins indicating the diversity of actin-binding domains (Figure 3A). Pkinase (including PK_Tyr_Ser-Thr family of tyrosine and serine-threonine protein kinase) was the most abundant domain family, followed by LIM, CH, SH3_1, PH, RRM_1, PDZ, Ank_2, and Myosin_head domain. PKinase was the most abundant Pfam clan, followed by P-loop_NTPase, SH3, PH, Beta-propeller, Zn_Beta_Ribbon, and E-set. Around sixteen Pfam clans have 100 or more AAPs (Figure 3B).

For the list of AAPs, we have tried to identify the actin-binding module. In most of the proteins, the actin-binding domain/motif has not been characterized (Supplementary Table S2).

### 3.3 Structural domains in AAPs

We identified 494 unique structural domains organized in 838 unique domain architectures (Supplementary Table S2). Similar to the Pfam domains, only thirteen are found in more than 40 proteins (Figure 3C). The most abundant protein domain superfamilies were P-loop containing nucleoside triphosphate hydrolases, protein kinase-like (PK-like), SH3-domain, PH domain-like, glucocorticoid receptor-like (DNA-binding domain), and calponin homology domain (CH domain). The protein domains belonged to 365 unique folds. Around twelve SCOP folds have 50 or more AAPs (Figure 3D). The most abundant Pfam folds were P-loop containing nucleoside triphosphate hydrolases, protein kinase-like (PK-like), SH3-like barrel, immunoglobulin-like beta-sandwich, PH domain-like barrel, and DNA/RNA-binding 3-helical bundle. All alpha were the most abundant, followed by all beta protein, alpha and beta proteins (a+b), and alpha and beta protein (a/b) class. This is consistent with the observation that most of the AAPs interact with actin using an alpha-helical motif (Dominguez and Holmes, 2011). Disorder content was observed in about 38% of the proteins, indicating that the AAPs might have other functions like protein–protein interactions.

### 3.4 CH domains and actin binding

The CH domain is an actin-binding domain that occurs in many AAPs. CH domain is common to the spectrin family of proteins, such as spectrin and nesprin, and the utrophin family of proteins, such as utrophin, dystrophin, and filamin (Yin et al., 2020). The CH domain is also known to bind to tubulin and signaling proteins to mediate cellular processes (Yin et al., 2020). CH domain is found in a wide variety of protein domain architectures, associating with calponin repeats, spectrin repeats, plakin repeats, E-set domains, glucocorticoid receptor-like (DNA-binding domain), EF-hand domain, RasGAP C-terminal domain, *etc*. (Korenbaum and Rivero, 2002).

Some of the domain combinations had a restricted phylogenetic distribution, such as the combination of an N-terminal EF-hand domain followed by CH domains. This combination is found in plastin proteins which are found only in chordates. Plastin is an actin cross-linking protein required for stereocilia formation in the inner ear and for the brush border assembly in the intestinal epithelium (Krey et al., 2016). However, not all CH domains can bind actin. In calponin-2 (gene name: CNN2), the actin-binding happens *via* a cryptic actin-binding site within the calponin repeats in the C-terminal region and not through the N-terminal CH domain. Similarly, in calponin-1 (gene name: CNN1), actin binding is mediated by both the CH domain and the cryptic site within the calponin repeat region (Gimona and Mital, 1998). This shows the versatility of actin-binding domains and motifs. The CH domain might have evolved to bind to other proteins and lost the ability to bind actin during the course of evolution.

### 3.5 Cellular localization

Roughly half of the proteins (1,381) were associated with the membrane-integral membrane proteins (308) and peripheral membrane proteins (224), and around 44% of the putative AAPs (1,101) were predicted to be cytoplasmic. Of the membrane localizing proteins, 111 had lipid modifications such as farnesylation (13), myristoylation (30), palmitoylation (38), and geranylgeranylation (22), and 12 proteins had more than one type of lipid modification (Supplementary Data S4).

### 3.6 Abundance of AAPs across tissues

We used the HPA database and UniProt database metadata to study the tissue abundance and enrichment of the AAPs. A total of 43 proteins were enriched only in 1 tissue (20 of these had high expression in the tissues). Roughly 98% of the AAPs were expressed across multiple tissue types, indicating the important functions of AAPs. A total of 1,306 AAPs were expressed in 2-5 tissues, while 114 proteins were expressed in >40 tissues. Most of the proteins had either “high” or “medium” levels of expression in the tissues (Supplementary Table S3).

### 3.7 Conservation of AAPs and their families

We identified the taxonomic distribution of the orthologs of the AAPs from the eggNOG database. With the exception of 5 AAPs which have identified orthologs in eukaryotes, most of the AAPs identified conserved orthologs across metazoa (Supplementary Table S4A). We also identified the taxonomic distribution of the Pfam domains associated with the AAPs (Supplementary Table S4B). Most of the domain families were conserved across eukaryotes, followed by metazoa, consistent with previous reports (Pollard, 2016). Many domain families were found to have orthologs in eukaryotes-bacteria-archaea or eukaryotes-bacteria lineages in Pfam.

### 3.8 Functional classification of AAPs

#### 3.8.1 GO term enrichment analysis

Out of the 2,482 AAPs, 2,424 were annotated with at least a single GO term from the UniProt database (2,399 for CC, 2,272 for MF, and 2,352 for BP) and categorized accordingly. We used the GO enrichment module from the ShinyGO web resource for the enrichment analysis.

**FIGURE 4:**
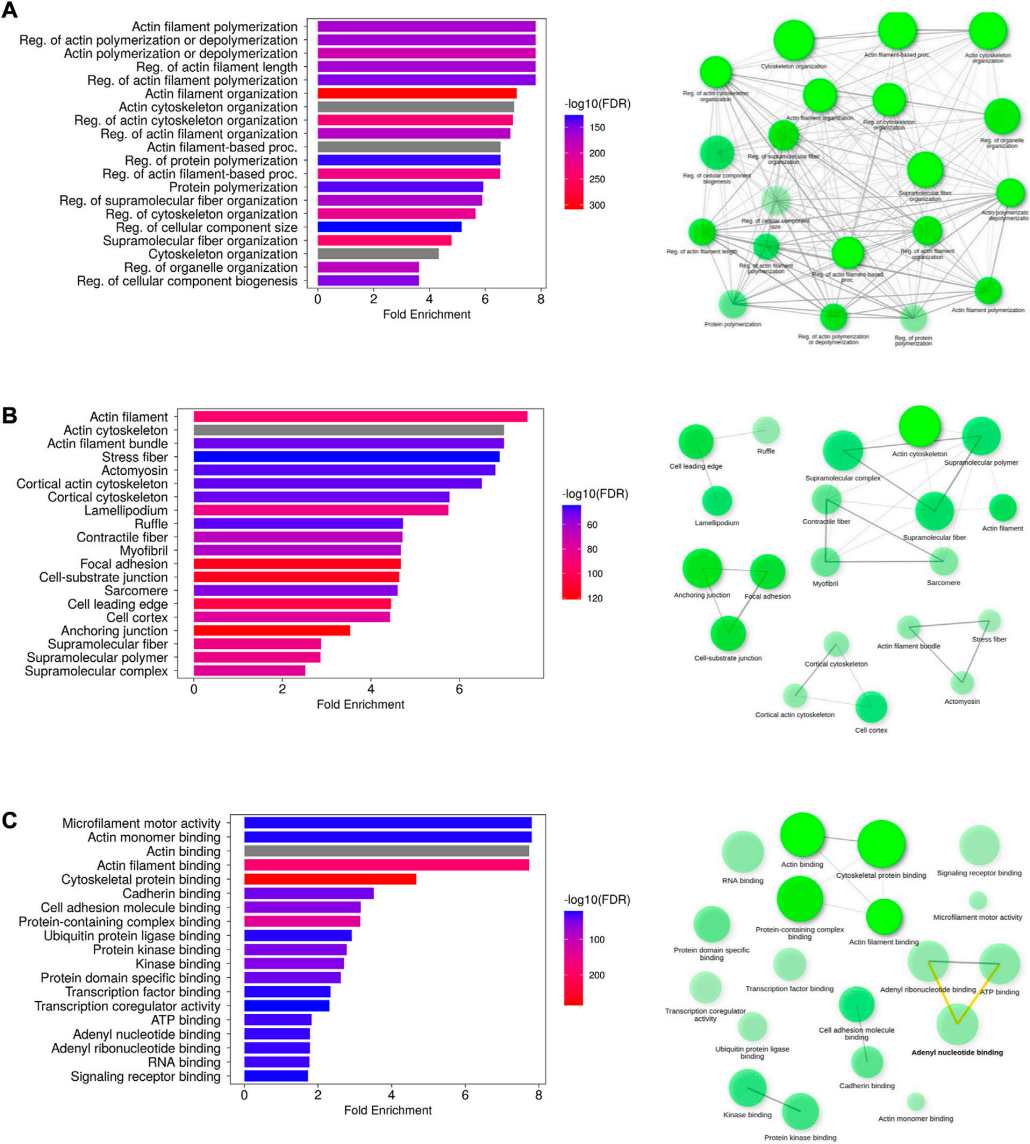
Functional analysis of putative AAPs. The enriched GO terms representing the biological process **(A)**, cellular component **(B)**, and molecular function **(C)** have been depicted as bar plots. The network diagrams indicate the interconnection between the GO terms in each category. The most enriched GO terms are actin filament polymerization/depolymerization (biological process), localized to the actin filament (cellular localization), and microfilament activity (molecular function).

The highly enriched actin-related biological processes were “actin cytoskeleton reorganization,” “actin polymerization or depolymerization,” and “regulation of actin filament length”. Additional enriched processes include “regulation of cellular component size,” “cell morphogenesis,” “plasma membrane-bound cell projection organization,” and “locomotion.” The cellular components encompassed cellular structures such as “actin filament,” “actin cytoskeleton,” “stress fiber,” “lamellipodium,” “ruffle,” “cell–cell junction,” “neuron projection,” and “plasma membrane region.” Muscle-related cellular locations such as “myosin complex,” “myofibril,” “sarcomere,” and “I band” were also enriched. Their molecular functions primarily involved “microfilament motor activity,” “actin monomer/filament binding,” and “structural constituent of cytoskeleton.” Protein and other molecule binding terms include “myosin binding,” “calmodulin binding,” SH3 domain binding,” “cadherin binding,” “GTPase binding,” “kinase binding,” “lipid binding,” “ATP binding,” and “adenyl ribonucleotide binding.” The results are presented in Figures 4A–C.

#### 3.8.2 COG-based analysis

We further classified the AAPs into functionally related clusters using the COG classification. COG includes 24 functional categories encompassing functions such as information storage and processing, cellular processes and signaling, metabolism, and poorly characterized. About 2,168 proteins were assigned to a single COG term, whereas 123 proteins were assigned to multiple COG terms (Supplementary Table S4A). A total of 190 proteins were assigned the COG term function unknown (S category). The most abundant categories were T (signal transduction mechanism), Z (cytoskeleton), K (transcription), O (post-translational modification), U (intracellular trafficking), and A (RNA processing and modification). Most of the AAPs were involved in cellular processes and signaling (1,406), followed by information storage and processing (651) and metabolism-related (185) functions (Supplementary Table S4C).

### 3.9 AAPs and human diseases

**FIGURE 5:**
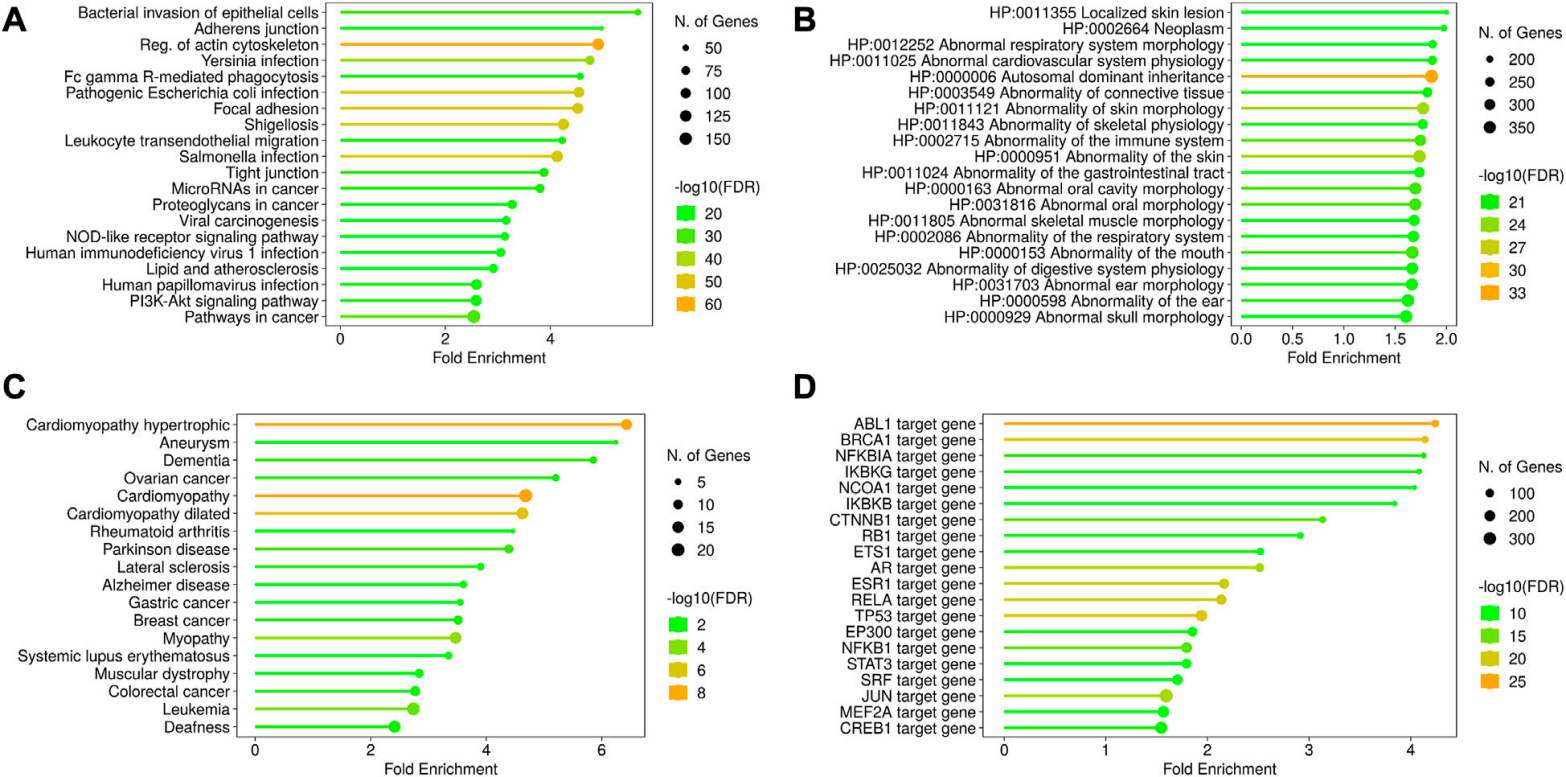
Disease ontology and gene regulation details of AAPs. The KEGG pathway enrichment shows the processes related to infection, cell structures, and other disorders in the cell **(A)**. The disease annotations have been derived from HPO **(B)** and OMIM **(C)** using the ShinyGO pipeline. These enriched annotations are for cardiomyopathy, abnormal morphology of tissues, and certain cancers. The transcription factors regulating the AAPs have been shown in **(D)**. The AAP list shows the enrichment of transcription factors regulating ABL1 and BRCA1 genes, denoting their roles in tumor suppression.

A total of 892 proteins were found to be associated with disease conditions using the DisGeNET webserver and the HPO resource (Supplementary Table S5). The terms related to the respiratory system, cardiovascular system, connective tissue, skeletal muscle, immune system, and autosomal dominant inheritance were enriched from the HPO resource, whereas the terms related to cardiomyopathy, aneurysm, dementia, Alzheimer’s and Parkinson’s disease, cancers, and myopathies were enriched from the OMIM resource. The enriched KEGG terms were associated with four broad classes of functions, such as infection, cell structure, cancer, and metabolism-related disorders. The infection-related functions included pathways related to intracellular bacteria and virus infection and immune response to infections (Figures 5A–D, Supplementary Figure 2A–E).

HPA pathology expression data suggested that 788 AAPs are associated with cancer. A total of 165 AAPs were found to be associated with more than one cancer type. ARHGAP26 and FAM53B were found associated with five cancer types, and PIP5K1C, FBP1, and CUL9 were found to be associated with four cancer types.

## 4 Discussion

We identified 32 human proteins containing actin domains; six of these were categorized as conventional actins, two as centractins, eleven as actin-related proteins, eight as actin-like proteins, and five as POTE ankyrins (keyword search from UniProt for actin domain). Among these proteins, conventional actins are major components of the cytoskeleton and can polymerize to form F-actin. We focused on the conventional actins and mined for their associated proteins using protein–protein interaction data from several databases and the PDB resource, actin-related GO terms, classical actin-binding domains, and the literature.

AAPs have been commonly classified and studied based on their regulatory effects on actin filaments and also based on functional and structural ABDs (Pollard, 2016). Actins are functionally conserved across vertebrates (Arora et al., 2023). ABDs are deeply conserved across eukaryotes, whereas actin and AAP orthologs are conserved across metazoa (Pollard, 2016). This hints toward the evolutionary radiation of AAPs with actin. The 2,482 human likely AAPs we identified contain a repertoire of 1824 Pfam domains and 494 structurally distinct domains. Among the ABD Pfam families, only 13 have more than 10 gene members; most families (1,176) have only one member. A common feature of the AAPs is the presence of disordered regions. On average, about 38% of the AAPs have disordered regions that cover 12% of the sequences.

Automated functional annotations, including the Gene Ontology project, integrate literature reports, database entries, and structural features. They might include proteins that were falsely assigned as participating in biological processes as inferred from homology. They might also exclude valid proteins owing to less evidence or the absence of annotation. Similarly, domain analysis might include some false positives, as all proteins that have an ABD might not bind to actin. Some of the proteins do not have a well-defined actin-binding domain, such as the Kelch repeat and BTB domain-containing protein 13 (UniProt id: C9JR72), Aquaporin-1 (UniProt: P29972), and septin 9 (Uniprot id: Q9UHD8) (Sasaki et al., 2014; Smith et al., 2015; de Winter et al., 2020). This could mean that there might be some proteins with actin-binding motifs which might not have been captured by our approach. The actin-binding motifs of at least two-thirds of the 2,482 RBPs are unknown. We are currently far from a comprehensive characterization of ABDs and AAPs. Even among the established large classes of ABDs, many individual members from the protein families have not been studied in detail, including SAB, FH2, villin headpiece domain, profilin, gelsolin, and the Kelch motif.

Most actin-binding protein families are found across eukaryotes, indicating that their ancestral genes were formed in the common eukaryotic ancestor or at the start point of the radiation of the eukaryotic kingdom (Dominguez and Holmes, 2011). This indicates that actin filament assembly regulation is one of the most conserved cellular processes. The interfamily relationships of actin AAPs might also be ancient. Some of the actin–AAP interactions are conserved across eukaryotes, from yeast to humans. This includes the interactions required to maintain a pool of G-actin, regulate filament assembly, transport cargo across the cell (Arp2/3, cofilin, formins, myosin, *etc*.), and the interactions required for eukaryotic translation and translation (eEF1A and IMPACT) (Goodson and Hawse, 2002; Sattlegger et al., 2014; Akram et al., 2020). However, there are differences in the copy number of some AAPs in multicellular organisms, such as formins (Gunning et al., 2015).

The structures of many AAPs are still lacking. However, we have tried to bridge the gap using sequence-based structure prediction methods. In some cases, the domains are common to more than one of the traditional families of proteins, establishing links between these families of proteins. In certain cases, actin-binding domains were seen to associate with different domains rendering different functions to the AAPs (Yin et al., 2020). This might have different effects on actin dynamics and regulation. Together, this has resulted in the diversity of domain architectures in AAPs. As the structures of more AAPs will be solved and deposited, more AAP structural domains and modules and their binding modes with actin might be identified.

## 5 Conclusion

A census of human AAPs is essential for the molecular and genetic understanding of the various functions of actins and their regulation. This catalog provides researchers with a curated resource to guide investigations of actins and their functions. Of the ∼20,500 protein-coding genes in humans, we determined that 7.5% are directly involved in actin regulation by binding to and/or processing actins, or by constituting essential components of AAPs.

AAPs are structurally and functionally diverse and include (Goodson and Hawse, 2002; Sattlegger et al., 2014; Akram et al., 2020) many distinct classes of ABDs. Indeed, the four most abundant ABDs accounted for only 10% of all AAPs in our census. Based on the GO term functional classification, we found that most of the AAPs were involved in actin filament binding and regulation, and some of them were involved in processes such as cellular organelle organization, locomotion, cell projection formation, and cell morphogenesis. The function-based categorization of AAPs can assist in the interpretation of disease phenotypes and mutations from patient sample data documented in databases and the literature. Most AAPs were ubiquitously expressed, and up to 12% of the total expressed protein-coding transcripts were encoded, indicating that AAPs are required in the cell in high protein copy number.

An analogous catalog that assesses the motifs/domains interacting with actins is still missing for some of the proteins. Such a catalog along with the solved crystal structures of the interactions showing the binding modes of actin would be a useful complementary document to this census.

## Data Availability

The datasets presented in this study can be found in online repositories. The names of the repository/repositories and accession can be found in the article/Supplementary Material. All codes are available at the following link: http://caps.ncbs.res.in/downloads/abp/. Any additional information required to re-analyse the data can be acquired from the authors.

## Supplementary material

Supplementary Figure 1 Image 1

Supplementary Figure 2a Image 2

Supplementary Figure 2b Image 3

Supplementary Figure 2c Image 4

Supplementary Figure 2d Image 5

Supplementary Figure 2e Image 6
