# Supplementary figures and images for: A census of actin-associated proteins in humans
Source: Front Cell Dev Biol. 2023 Apr 28;11:1168050. doi: 10.3389/fcell.2023.1168050 (PMC10175787; doi:10.3389/fcell.2023.1168050)

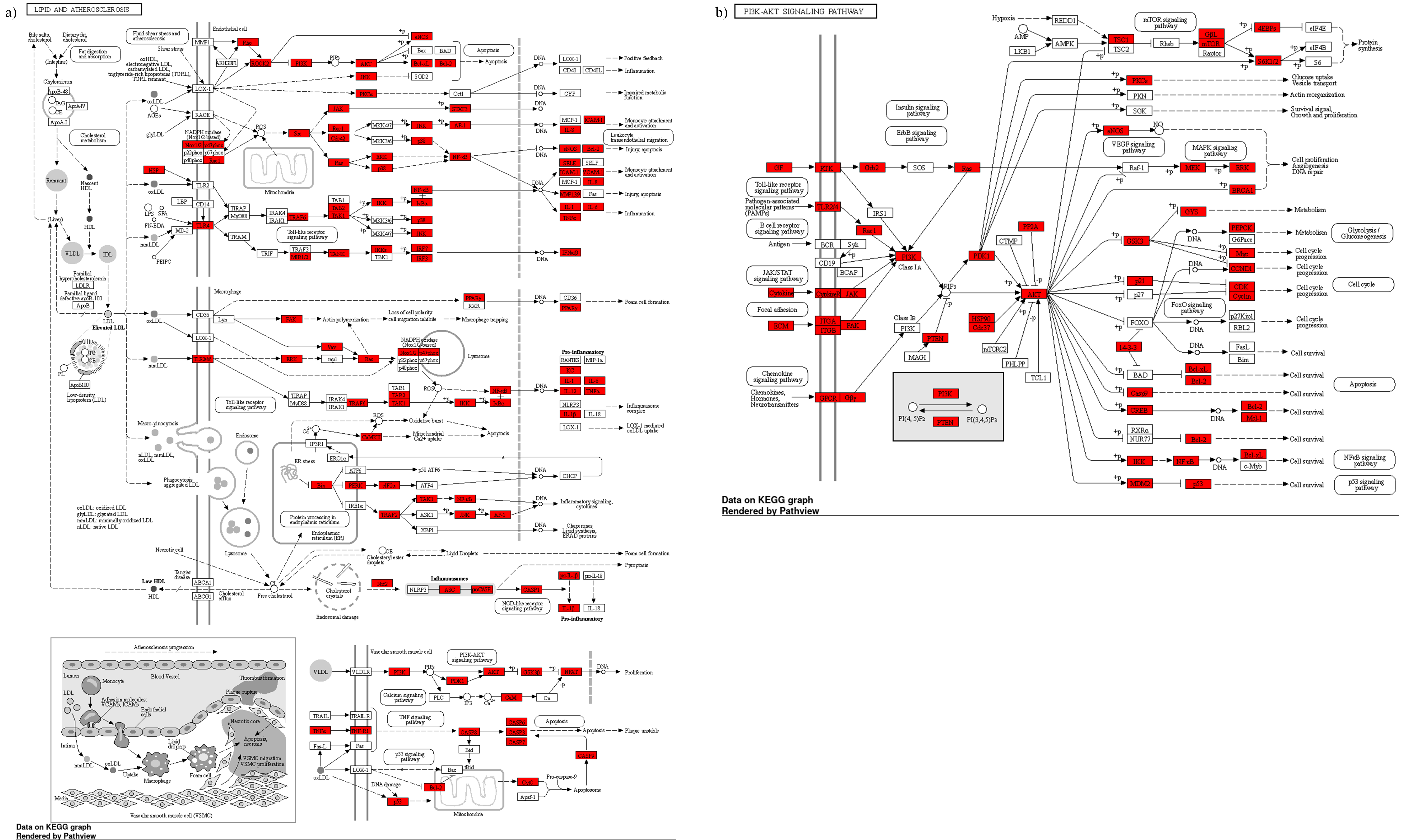

Supplement: Supplementary file 3 [file Image6.tif]

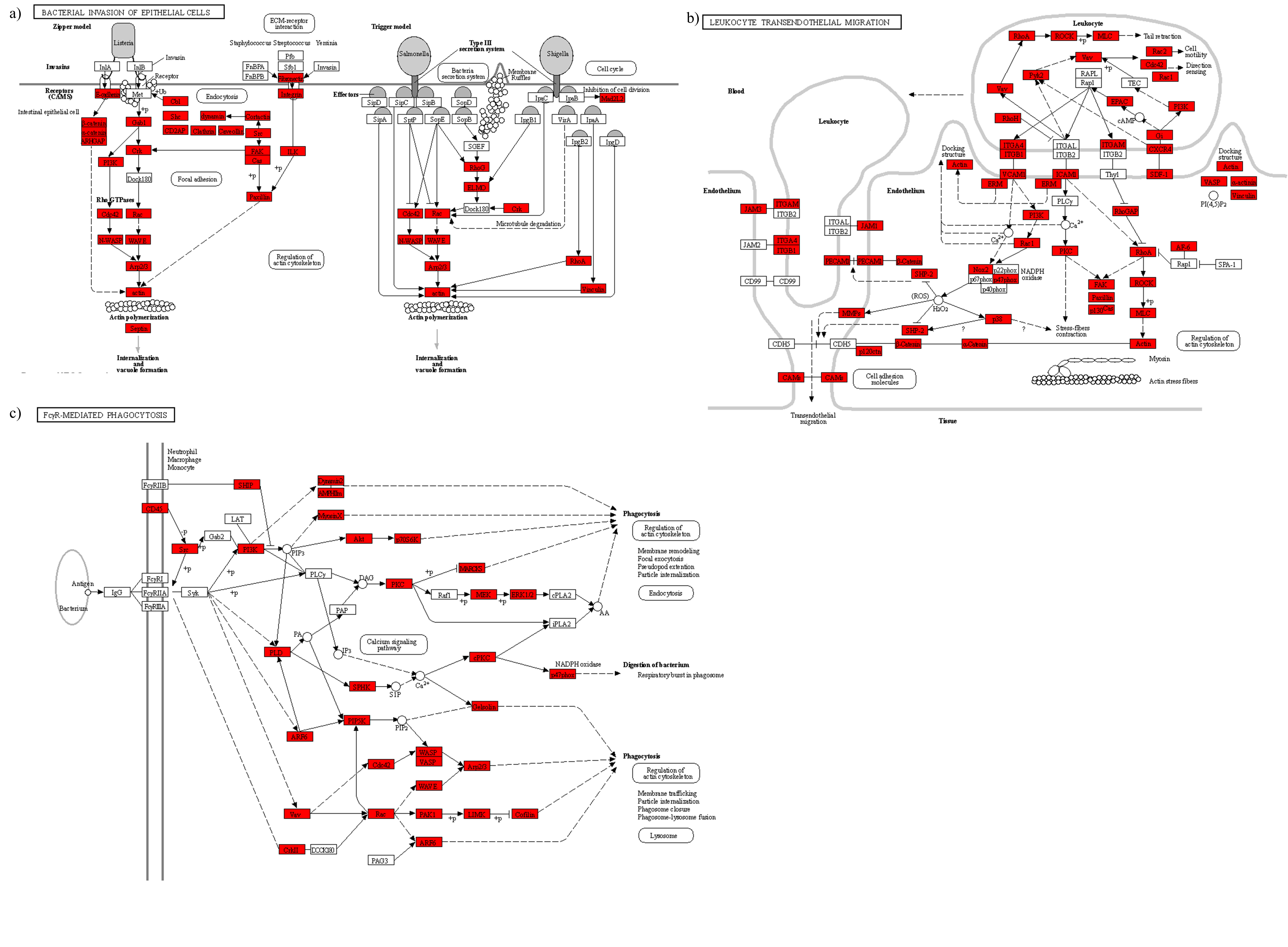

Supplement: Supplementary file 5 [file Image3.tif]

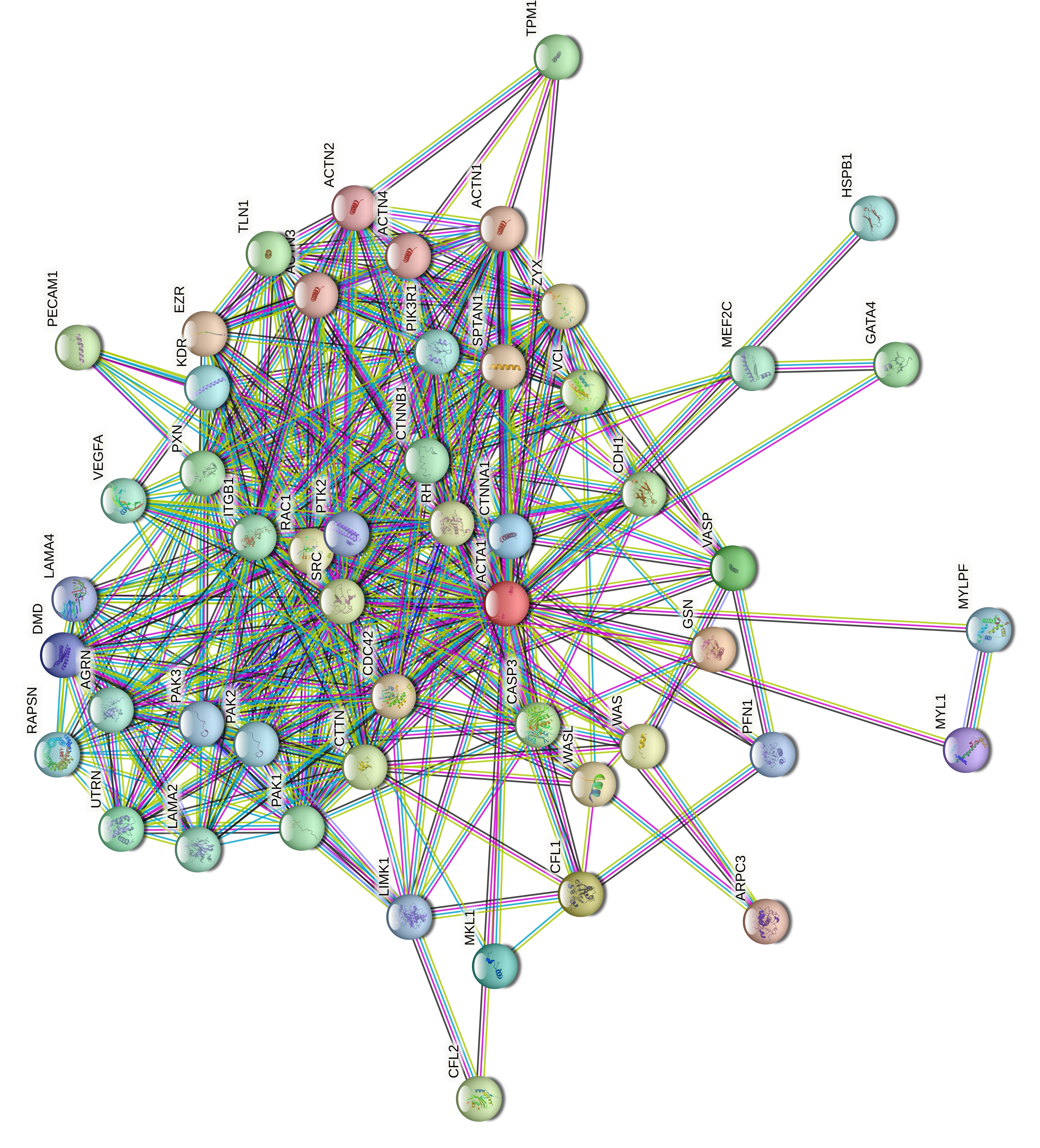

Supplement: Supplementary file 11 [file Image1.png]
