# Supplementary material for: A census of actin-associated proteins in humans
Source: Front Cell Dev Biol. 2023 Apr 28;11:1168050. doi: 10.3389/fcell.2023.1168050 (PMC10175787; doi:10.3389/fcell.2023.1168050)
Supplement: Supplementary file 17 [file DataSheet1.docx]

**A census of actin- associated proteins in humans**

Iyer Meenakshi S^1^, Madan Rao^1^, Satyajit Mayor^1^, Ramanathan Sowdhamini^1*^

^*^Correspondence: Corresponding author: mini@ncbs.res.in

1 National Centre for Biological Sciences, TIFR, Bangalore, India

1. **Supplementary Results:**

**Isoforms of AAPs**

We used the Pfam protein-domain hidden Markov models (HMMs) to search the human proteome, comprising 20,386 protein-coding genes (21,989 additional isoforms), followed by a stringent filtering criteria to assign domains to sequences. We identified 243 AAPs (727 isoforms) from the literature-based Pfam domain assignment method. We also used the GO annotations of proteins in UniProt database to identify 772 AAPs (2178 isoforms). Additionally, we used Pfam domain GO annotations (87 AAPs with 191 isoforms) and PDB GO annotations (27 AAPs with 61 isoforms) to identify protein domains with putative AAP function (**Supplementary Table 2**).

**Pfam DA of the isoforms**

The AAPs we identified contain a repertoire of 1824 distinct Pfam (functional) domains in 1574 distinct domain architectures (1837 domains with 2109 domain architectures including isoforms) (**Supplementary Table 3**).

1. **Supplementary Data:**

**Supplementary Data 1: List of actins identified from PPI databases**

The list of AAPs identified for alpha actin 1 (a), alpha actin 2 (b), alpha actin 3 (c), alpha cardiac actin (d), beta actin (e) and gamma actin (f) using the different PPI databases listed in **Table 2**.

**Supplementary Data 2: List of AAPs identified through different methods**

The list of AAPs identified using different approaches such as PPI databases (a), Pfam domain annotation (b), GO metadata (c) and literature (d).

**Supplementary Data 3**: Interacting partners for the protein-protein interaction network of skeletal muscle actin (alpha actin 1)

The UniProt protein names, description and interaction scores of the interacting partners of ACTN1 protein (alpha actin 1) has been provided. Only the highest confidence interactions (interaction score > 0.9) were retained. Only top 50 interactions were chosen for image clarity.

**Supplementary Data 4: AAPs sorted according to their cellular localisation**

We broadly sorted the AAPs into cytoplasmic, integral (cell membrane and organelle membrane), peripheral membrane and adapter proteins.

1. **Supplementary Tables:**

**Supplementary Table 1a: List of Pfam family actin-binding domains identified through literature**

The Pfam family name, the family identifier and the corresponding reference for actin binding properties of the domain families has been reported.

| **Pfam family ID** | **Pfam family name** | **Pfam family ID** | **Reference** |
| --- | --- | --- | --- |
| Act-Frag_cataly | Actin-fragmin kinase, catalytic | PF09192 | Steinbacher S, Hof P, Eichinger L, et al. The crystal structure of the Physarum polycephalum actin-fragmin kinase: an atypical protein kinase with a specialized substrate-binding domain. EMBO J. 1999;18(11):2923-2929. doi:10.1093/emboj/18.11.2923 |
| Annexin | Annexin | PF00191 | Hayes MJ, Rescher U, Gerke V, Moss SE. Annexin-actin interactions. Traffic. 2004;5(8):571-576. doi:10.1111/j.1600-0854.2004.00210.x |
| ASD1 | Apx/Shroom domain ASD1 | PF08688 | Dietz ML, Bernaciak TM, Vendetti F, Kielec JM, Hildebrand JD. Differential actin-dependent localization modulates the evolutionarily conserved activity of Shroom family proteins. J Biol Chem. 2006;281(29):20542-20554. doi:10.1074/jbc.M512463200 |
| CAP_C | Adenylate cyclase associated (CAP) C terminal | PF08603 | Bertling E, Hotulainen P, Mattila PK, Matilainen T, Salminen M, Lappalainen P. Cyclase-associated protein 1 (CAP1) promotes cofilin-induced actin dynamics in mammalian nonmuscle cells. Mol Biol Cell. 2004;15(5):2324-2334. doi:10.1091/mbc.e04-01-0048 |
| CH | Calponin homology (CH) domain | PF00307 | Stradal T, Kranewitter W, Winder SJ, Gimona M. CH domains revisited. FEBS Lett. 1998;431(2):134-137. doi:10.1016/s0014-5793(98)00751-0 |
| Cofilin_ADF | Cofilin/tropomyosin-type actin-binding protein | PF00241 | Lappalainen P, Kessels MM, Cope MJ, Drubin DG. The ADF homology (ADF-H) domain: a highly exploited actin-binding module. Mol Biol Cell. 1998;9(8):1951-1959. doi:10.1091/mbc.9.8.1951 |
| F_actin_bind | F-actin binding | PF08919 | Hantschel O, Wiesner S, Güttler T, et al. Structural basis for the cytoskeletal association of Bcr-Abl/c-Abl. Mol Cell. 2005;19(4):461-473. doi:10.1016/j.molcel.2005.06.030 |
| F-actin_cap_A | F-actin capping protein alpha subunit | PF01267 | Maruyama K, Kurokawa H, Oosawa M, et al. Beta-actinin is equivalent to Cap Z protein. J Biol Chem. 1990;265(15):8712-8715. |
| F_actin_cap_B | F-actin capping protein, beta subunit | PF01115 | Maruyama K, Kurokawa H, Oosawa M, et al. Beta-actinin is equivalent to Cap Z protein. J Biol Chem. 1990;265(15):8712-8715. |
| Fascin | Fascin domain | PF06268 | Bryan J, Edwards R, Matsudaira P, Otto J, Wulfkuhle J. Fascin, an echinoid actin-bundling protein, is a homolog of the Drosophila singed gene product. Proc Natl Acad Sci U S A. 1993;90(19):9115-9119. doi:10.1073/pnas.90.19.9115 |
| FH2 | Formin Homology 2 Domain | PF02181 | Shimada A, Nyitrai M, Vetter IR, et al. The core FH2 domain of diaphanous-related formins is an elongated actin binding protein that inhibits polymerization. Mol Cell. 2004;13(4):511-522. doi:10.1016/s1097-2765(04)00059-0 |
| Filamin | Filamin/ABP280 repeat | PF00630 | Fucini P, Renner C, Herberhold C, Noegel AA, Holak TA. The repeating segments of the F-actin cross-linking gelation factor (ABP-120) have an immunoglobulin-like fold. Nat Struct Biol. 1997;4(3):223-230. doi:10.1038/nsb0397-223 |
| Gelsolin | Gelsolin repeat | PF00626 | Weeds AG, Gooch J, Pope B, Harris HE. Preparation and characterization of pig plasma and platelet gelsolins. Eur J Biochem. 1986;161(1):69-76. doi:10.1111/j.1432-1033.1986.tb10125.x |
| LIM | LIM domain | PF00412 | Sun X, Phua DYZ, Axiotakis L Jr, et al. Mechanosensing through Direct Binding of Tensed F-Actin by LIM Domains. Dev Cell. 2020;55(4):468-482.e7. doi:10.1016/j.devcel.2020.09.022 |
| Myosin_head | Myosin head (motor domain) | PF00063 | Rayment I, Holden HM, Whittaker M, et al. Structure of the actin-myosin complex and its implications for muscle contraction. Science. 1993;261(5117):58-65. doi:10.1126/science.8316858 |
| Prefoldin | Prefoldin subunit | PF02996 | Millán-Zambrano G, Chávez S. Nuclear functions of prefoldin. Open Biol. 2014 Jul;4(7):140085. doi: 10.1098/rsob.140085. PMID: 25008233; PMCID: PMC4118604. |
| Prefoldin_2 | Prefoldin subunit | PF01920 | Millán-Zambrano G, Chávez S. Nuclear functions of prefoldin. Open Biol. 2014 Jul;4(7):140085. doi: 10.1098/rsob.140085. PMID: 25008233; PMCID: PMC4118604. |
| Prefoldin_3 | Prefoldin subunit | PF13758 | Millán-Zambrano G, Chávez S. Nuclear functions of prefoldin. Open Biol. 2014 Jul;4(7):140085. doi: 10.1098/rsob.140085. PMID: 25008233; PMCID: PMC4118604. |
| Profilin | Profilin | PF00235 | Yarmola EG, Bubb MR. Profilin: emerging concepts and lingering misconceptions. Trends Biochem Sci. 2006;31(4):197-205. doi:10.1016/j.tibs.2006.02.006 |
| Synapsin | Synapsin, N-terminal domain | PF02078 | Petrucci TC, Morrow JS. Actin and tubulin binding domains of synapsins Ia and Ib. Biochemistry. 1991;30(2):413-422. doi:10.1021/bi00216a016 |
| Synapsin_N | Synapsin N-terminal | PF10581 | Petrucci TC, Morrow JS. Actin and tubulin binding domains of synapsins Ia and Ib. Biochemistry. 1991;30(2):413-422. doi:10.1021/bi00216a016 |
| Synapsin_C | Synapsin, ATP binding domain | PF02750 | Petrucci TC, Morrow JS. Actin and tubulin binding domains of synapsins Ia and Ib. Biochemistry. 1991;30(2):413-422. doi:10.1021/bi00216a016 |
| Thymosin | Thymosin beta-4 family | PF01290 | Xue B, Leyrat C, Grimes JM, Robinson RC. Structural basis of thymosin-β4/profilin exchange leading to actin filament polymerization. Proc Natl Acad Sci U S A. 2014;111(43):E4596-E4605. doi:10.1073/pnas.1412271111 |
| Tropomodulin | Tropomodulin | PF03250 | Kostyukova AS. Capping complex formation at the slow-growing end of the actin filament. Biochemistry (Mosc). 2008;73(13):1467-1472. doi:10.1134/s0006297908130075 |
| Tropomyosin | Tropomyosin | PF00261 | Lewis WG, Smillie LB. The amino acid sequence of rabbit cardiac tropomyosin. J Biol Chem. 1980;255(14):6854-6859. |
| EVH2 | Ena/VASP homology 2 | - | Gentry, B.S., van der Meulen, S., Noguera, P. et al. Multiple actin binding domains of Ena/VASP proteins determine actin network stiffening. Eur Biophys J 41, 979–990 (2012). https://doi.org/10.1007/s00249-012-0861-1 |
| Vinculin | Vinculin family | PF01044 | Herrenknecht K, Ozawa M, Eckerskorn C, Lottspeich F, Lenter M, Kemler R. The uvomorulin-anchorage protein alpha catenin is a vinculin homologue. Proc Natl Acad Sci U S A. 1991;88(20):9156-9160. doi:10.1073/pnas.88.20.9156 |
| VHP | Villin headpiece domain | PF02209 | Vardar D, Chishti AH, Frank BS, et al. Villin-type headpiece domains show a wide range of F-actin-binding affinities. Cell Motil Cytoskeleton. 2002;52(1):9-21. doi:10.1002/cm.10027 |
| WH2 | WH2 motif | PF02205 | Machesky LM, Insall RH. Scar1 and the related Wiskott-Aldrich syndrome protein, WASP, regulate the actin cytoskeleton through the Arp2/3 complex. Curr Biol. 1998;8(25):1347-1356. doi:10.1016/s0960-9822(98)00015-3 |
| ERM_C | Ezrin/radixin/moesin family C terminal | PF00769 | Tsukita S, Yonemura S, Tsukita S. ERM proteins: head-to-tail regulation of actin-plasma membrane interaction. Trends Biochem Sci. 1997;22(2):53-58. doi:10.1016/s0968-0004(96)10071-2 |

**Supplementary Table 1b: List of Pfam domains with actin-binding/localising GO terms**

The annotations were derived from the Pfam2GO mapping provided in the Pfam database. The Pfam id and the corresponding actin-binding function-related GO terms have been indicated.

| **GO term** | **GO ID** | **Pfam family ID** | **Pfam family** |
| --- | --- | --- | --- |
| actin binding | GO0003779 | PF00235 | Profilin |
| actin cortical patch | GO0030479 | PF17096 | AIM3 |
| actin cytoskeleton | GO0015629 | PF04045 | P34-Arc |
| actin cytoskeleton organization | GO0030036 | PF06371 | Drf_GBD |
| actin cytoskeleton reorganization | GO0031532 | PF07487 | SopE_GEF |
| actin filament binding | GO0051015 | PF01044 | Vinculin |
| actin filament bundle organization | GO0061572 | PF15068 | FAM101 |
| actin filament organization | GO0007015 | PF01290 | Thymosin |
| actin filament polymerization | GO0030041 | PF05856 | ARPC4 |
| actin filament reorganization | GO0090527 | PF16671 | ACD |
| actin monomer binding | GO0003785 | PF01290 | Thymosin |
| Arp2/3 complex-mediated actin nucleation | GO0034314 | PF04045 | P34-Arc |
| barbed-end actin filament capping | GO0051016 | PF01115 | F_actin_cap_B |
| cortical actin cytoskeleton organization | GO0030866 | PF04382 | SAB |
| F-actin capping protein complex | GO0008290 | PF01115 | F_actin_cap_B |
| negative regulation of actin nucleation | GO0051126 | PF10574 | UPF0552 |
| pointed-end actin filament capping | GO0051694 | PF03250 | Tropomodulin |
| regulation of actin filament polymerization | GO0030833 | PF04045 | P34-Arc |
| regulation of Arp2/3 complex-mediated actin nucleation | GO0034315 | PF15745 | AP1AR |

**Supplementary Table 1c: GO terms associated with actin-binding/localisation**

These GO terms were derived from the human proteome GO annotation terms derived from UniProt metadata.

**Supplementary Table 2: Pfam domain, domain architecture and actin binding motif annotation of the AAPs**

A master table listing the reviewed human AAPs, Pfam domain architecture, SCOP structural domain architecture and actin binding motif annotation. The Pfam clan-level detail, SCOP folds, SCOP class and disorder content in the proteins has also been provided.

**Supplementary Table 3: Tissue abundance of AAPs derived from HPA database**

The distribution of AAPs across several tissues has been derived from the HPA database.

**Supplementary Table 4a-b: Taxonomic distribution of AAP orthologs and Pfam domains in AAPs and COG classification of AAPs**

The orthologs of the AAPs and their taxonomic distribution were derived from eggnog database (a). The Pfam domain taxonomic distribution details were derived from the Pfam database taxonomic distribution page (b). The eggnog database also provides COG category annotation of the AAPs. Some of the proteins have multiple COG category annotations, we derived the number of AAPs corresponding to each COG single letter annotation (c).

**Supplementary Table 4c: COG category annotation of the AAPs**

| **COG single letter code** | **Functional term** | **#AAPs** |
| --- | --- | --- |
| **INFORMATION STORAGE AND PROCESSING** | | |
| J | Translation, ribosomal structure and biogenesis | **68** |
| A | RNA processing and modification | **112** |
| K | Transcription | **342** |
| L | Replication, recombination and repair | **58** |
| B | Chromatin structure and dynamics | **71** |
| **CELLULAR PROCESSES AND SIGNALING** | | |
| D | Cell cycle control, cell division, chromosome partitioning | **59** |
| Y | Nuclear structure | **8** |
| V | Defense mechanisms | **17** |
| T | Signal transduction mechanisms | **460** |
| M | Cell wall/membrane/envelope biogenesis | **3** |
| N | Cell motility | **6** |
| Z | Cytoskeleton | **451** |
| W | Extracellular structures | **60** |
| U | Intracellular trafficking, secretion, and vesicular transport | **150** |
| O | Posttranslational modification, protein turnover, chaperones | **192** |
| **METABOLISM** | | |
| C | Energy production and conversion | **61** |
| G | Carbohydrate transport and metabolism | **24** |
| E | Amino acid transport and metabolism | **7** |
| F | Nucleotide transport and metabolism | **18** |
| H | Coenzyme transport and metabolism | **10** |
| I | Lipid transport and metabolism | **23** |
| P | Inorganic ion transport and metabolism | **38** |
| Q | Secondary metabolites biosynthesis, transport and catabolism | **4** |
| **POORLY CHARACTERIZED** | | |
| R | General function prediction only | **-** |
| S | Function unknown | **190** |

**Supplementary Table 5: Human disease association data of the AAPs derived from DisGeNET, HPO and HPA pathology datasets**

The disease ontology data has been concatenated from several resources such as DisGeNET, HPO and HPA. HPA provides oncology-related metadata for proteins associated with cancers. Whereas, the HPO and DisGeNET database provide data for several disorders and syndromes.

1. **Supplementary Figures**

**Supplementary Figure 1: PPI network of ACTA1 (skeletal muscle actin 1)**

The protein interaction network of alpha actin 1 (skeletal muscle actin) with highest confidence of interaction (interaction score > 0.9) derived from the STRING database has been depicted. We have shown only 50 interacting partners for clarity. The proteins are shown as circles (nodes) and the connecting lines depict the interactions (edges). The filled circles proteins with solved/predicted structures, proteins without solved or predicted structures are shown as empty circles. The connecting lines of different colours denote different sources of annotation, cyan: from curated databases, pink: experimentally determined, yellow: text-mining, black: co-expression, grey: protein homology.

**Supplementary Figure 2: KEGG pathway analysis**

The KEGG terms associated with AAPs fell into broadly five categories viz. interaction of intracellular bacteria with host proteins (a), host response to infection (b), cell structure related (c), cancer (d) and metabolism (e).
